# Comparative Immunological Study in Mice of Inactivated Influenza Vaccines Used in the Russian Immunization Program

**DOI:** 10.3390/vaccines8040756

**Published:** 2020-12-12

**Authors:** Andrei Shanko, Marina Shuklina, Anna Kovaleva, Yana Zabrodskaya, Inna Vidyaeva, Aram Shaldzhyan, Artem Fadeev, Alexander Korotkov, Marina Zaitceva, Liudmila Stepanova, Liudmila Tsybalova, Larisa Kordyukova, Anton Katlinski

**Affiliations:** 1 Research and Development Department, FORT LLC, 119435 Moscow, Russia; 2 N. F. Gamaleya Federal Research Center for Epidemiology and Microbiology, Ivanovsky Institute of Virology, 123098 Moscow, Russia; 3 WHO National Influenza Center, Smorodintsev Research Institute of Influenza, 197376 Saint-Petersburg, Russia; marina.shuklina@influenza.spb.ru (M.S.); anna.kovaleva@influenza.spb.ru (A.K.); yana.zabrodskaya@influenza.spb.ru (Y.Z.); inna.vidyaeva@influenza.spb.ru (I.V.); aram.shaldzhyan@influenza.spb.ru (A.S.); artem.fadeev@influenza.spb.ru (A.F.); lex9268@mail.ru (A.K.); marina.zaitceva@influenza.spb.ru (M.Z.); liudmila.stepanova@influenza.spb.ru (L.S.); sovet@influenza.spb.ru (L.T.); 4 Peter the Great Saint-Petersburg Polytechnical University, 194064 Saint-Petersburg, Russia; 5 Petersburg Nuclear Physics Institute named by B. P. Konstantinov of National Research Center “Kurchatov Institute”, 188300 Gatchina, Russia; 6 Belozersky Institute of Physico-Chemical Biology, Lomonosov Moscow State University, 119991 Moscow, Russia; kord@belozersky.msu.ru; 7 Board Member, FORT LLC, 119435 Moscow, Russia; info@fort-bt.ru

**Keywords:** inactivated influenza vaccines, immunogenicity, protection, HA-antibody response, NA-antibody response, SRD, HAI assay, MN assay, ELISA, mass spectrometry

## Abstract

A series of commercial inactivated influenza vaccines (IIVs) used in the Russian National Immunization Program were characterized to evaluate their protective properties on an animal model. Standard methods for quantifying immune response, such as hemagglutination inhibition (HAI) assay and virus neutralization (VN) assay, allowed us to distinguish the immunogenic effect of various IIVs from that of placebo. However, these standard approaches are not suitable to determine the role of various vaccine components in immune response maturation. The expanded methodological base including an enzyme-linked immunosorbent assay (ELISA) and a neuraminidase ELISA (NA-ELISA) helped us to get wider characteristics and identify the effectiveness of various commercial vaccines depending on the antigen content. Investigations conducted showed that among the IIVs tested, Ultrix^®,^ Ultrix^®^ Quadri and VAXIGRIP^®^ elicit the most balanced immune response, including a good NA response. For Ultrix^®^, Ultrix^®^ Quadri, and SOVIGRIPP^®^ (FORT LLC), the whole-virus specific antibody subclass IgG1, measured in ELISA, seriously prevailed over IgG2a, while, for VAXIGRIP^®^ and SOVIGRIPP^®^ (NPO Microgen JSC) preparations, the calculated IgG1/IgG2a ratio was close to 1. So, the immune response varied drastically across different commercial IIVs injected in mice.

## 1. Introduction

Despite the successes recorded in the fight against viral diseases, influenza remains one of the most virulent respiratory infections, while vaccination remains the only reliable method to contain it [1]. Given the antigenic drift in influenza virus, the World Health Organization (WHO) annually updates its guidelines on changes in seasonal influenza virus (IV) strain composition, most of which are inactivated split egg-based influenza vaccines (IIVs) consisting of 3 to 4 strains, circulating in the Northern and Southern hemispheres [2,3]. The main methods for assessing the efficacy of IIVs, which are well known and described in the regulatory formularies of WHO, EMA, and regional regulatory bodies, somehow include assays for the main IV antigen, i.e., hemagglutination inhibition (HAI) assay and the virus neutralization (VN) assay being used nowadays in micro-plate format and called the micro-neutralization (MN) assay [4,5]. The HAI assay is based on the ability of antibodies contained in immunized animal or human serum to inhibit red blood cell (RBC) agglutination, triggered by influenza hemagglutinin. According to the standards on anti-IV correlate of protection, a HAI titer of over 40% is associated with the protection level at which there is less than a 50% chance of infection. Despite the HAI being accepted as the gold standard, the assay shows some limitations, such as its low sensitivity to influenza B virus, dependence on RBC source, receptor destroying enzyme (RDE), and associated lab-specific variability of assay results [6]. The MN method draws on the measurement of neutralizing antibodies in paired samples of control and test sera of immunized animals or humans for inhibition of IV reproduction on Madin-Darby Canine Kidney (MDCK) cell culture. The main limitation of MN assay, apart from the need to work with live infectious IVs, which requires a defined biosafety level, is inter-laboratory variability of assay results due to the lack of common testing protocols, and inconsistencies in the final results. To date, MN assay parameters have not yet been worked out to correlate with the anti-IV protective effect. So, they are determined in each individual setting versus control. In addition to HAI and MN assays, the traditional ELISA is used to determine not only the general antibody titers, but also those of the Ig subclasses, and for various antigen targets, e.g., IV neuraminidase [7]. Apart from the high capacity available to standardize all reagents and primary and secondary antibodies, ELISA is suitable for high-throughput analysis and deep automation. All the aforesaid methods determine—in different ways—the immunological effect of a past disease or vaccination, and characterize various antibody types, and are all the more important in assessing the efficacy of vaccines from different manufacturers.

Apart from hemagglutinin, another important IV surface antigen is neuraminidase (NA), a tetrameric glycoprotein with enzymatic activity with respect to sialic acid and generally responsible for virion release from a damaged cell [8]. The NA immune response level is generally ignored by modern vaccine manufacturers, although there is a growing number of voices in support of the view that NA is also an important component of vaccination strategies [7,9,10]. Besides, it has been shown that the induction of NA-specific immunity in animals facilitated cross-protection against IV within the subtype [11,12]. This NA cross-protective effect was also shown in humans [13], thus proving yet again the relevance of determining immune response to NA during vaccination.

It’s known that the humoral immune response mediated by B cells and the antibody pool produced by these cells attack pathogens directly, while cellular immunity is created by T cells targeting the damaged body cells. The T cells, carrying CD4+ receptors, are divided into two types: T helper type 1 (Th1) lymphocytes, which secrete interleukin-2 (IL-2) and interferon-γ (IFN-γ), and T helper type 2 (Th2), which secrete IL-4, IL-5, IL-9, IL-10, and IL-13 [14]. The Th1-produced cytokines facilitate type 1 immune response and enhance the phagocytic activity of lymphocytes. Cytokines secreted by Th2 cells after antigen exposure trigger and maintain humoral immune response, and are characterized by a high antibody level [15,16]. The vaccine-induced IgG antibody distribution also indicates the immune response type, because IgG1 in mice, as believed, flags a Th2 response, whereas IgG2a indicates more the Th1 profile.

This paper aims at evaluating the specific activity of and the post‑vaccination immune response in animal models to split-virus IIVs, both trivalent and quadrivalent, approved for use in Russia under the Russian National Immunization Program for 2019/2020. In this regard, for a complete assessment of immunogenicity, we expanded the primary assay list to include, apart from HAI and MN assays, the ELISA and NA- enzyme-linked immunosorbent assay (ELISA) assays and determination of titers of IgGs and their specific subclasses, IgG1 and IgG2a. The protective potency of the IIVs under investigation was assessed based on the lethal IV model by estimating the count of dead animals, the timing of their death, body mass change, as well as determining the virus reproduction rate in animal lungs on day 3 after infection with IV strains on the vaccine list. 

## 2. Materials and Methods Used

### 2.1. Inactivated Split-Virion Influenza Vaccines (IIVs)

All the inactivated split-virion influenza vaccines (IIVs) studied were produced by propagating influenza viruses in chicken embryos. Both trivalent inactivated influenza vaccines (IIV3) and quadrivalent inactivated influenza vaccines (IIV4) were investigated and are listed below:

- U3—Ultrix^®^ (IIV3, FORT LLC, Russia);
- U4—Ultrix^®^ Quadri (IIV4, FORT LLC, Russia);
- SGF—SOVIGRIPP^®^ (IIV3, FORT LLC, Russia);
- SGU—SOVIGRIPP^®^ (IIV3, NPO Microgen JSC, Russia);
- VG—VAXIGRIP^®^ (IIV3, Sanofi Pasteur C.A., France).

The strain composition of all vaccines was consistent with WHO guidelines on the 2019–2020 seasonal influenza vaccines to be used in the Northern hemisphere, i.e., the trivalent vaccines:

- Influenza A virus (H1N1): A/Brisbane/02/2018 (H1N1)pdm09-like virus (A/Brisbane/02/2018, IVR-190);
- Influenza A virus (H3N2): A/Kansas/14/2017 (H3N2)-like virus (A/Kansas/14/2017, NYMC X-327);
- Influenza B virus (Victoria lineage): B/Colorado/06/2017-like strain (B/Maryland/15/2016, NYMC BX-69A).

An additional strain for the quadrivalent vaccines:

- Influenza B virus (Yamagata lineage): B/Phuket/3073/2013-like virus (B/Phuket/3073/2013 wild type).

U3, U4, and VG vaccines are free of adjuvants and preservatives. SGU and SGF vaccines contain SOVIDON^TM^ synthetic polymer (a copolymer of 2-methyl-5-vinylpyridine and N‑vinylpyrrolidone) –500 µg per dose (0.5 mL). Vaccine manufacturers claim that these polymers act as adjuvants. The SGU vaccine also contains thimerosal (merthiolate) as a preservative at 50 µg per dose (0.5 mL).

### 2.2. Viruses and Antigens, Antisera

The live influenza viruses were seasonal influenza strains obtained from FORT LLC: A/Brisbane/02/2018, IVR-190; A/Kansas/14/2017, NYMC X-327; B/Maryland/15/2016, NYMC BX-69A (Victoria lineage); B/Phuket/3073/2013 (Yamagata lineage). All the viruses used were egg-grown. The virus antigens were seasonal influenza strains obtained from NIBSC: influenza antigen A/Brisbane/02/2018 (IVR-190) (H1N1) NIBSC code:18/238 (HA 48 µg/mL); influenza antigen A/Kansas/14/2017 (NYMC X-327) (H3N2) NIBSC code:19/104 (HA 67 µg/mL); influenza antigen B/Maryland/15/2016 (NYMC BX-69A) NIBSC code:18/104 (HA 69 µg/mL); influenza antigen B/Phuket/3073/2013 NIBSC code:16/158 (HA 60 µg/mL). The antisera were obtained from NIBSC: influenza anti-A/Brisbane/02/2018-like (H1N1) HA serum (NIBSC code:19/102), influenza anti-A/Kansas/14/2017-like (H3N2) HA serum (NIBSC code:19/110), influenza anti-B/Colorado/06/2017-like HA serum (NIBSC code:18/170), and influenza anti-B/Phuket/3073/2013 HA serum (NIBSC code:15/150).

Live viruses were used for MN and HAI assays and a sublethal challenge; inactivated viruses were used for ELISA; the antigen preparations, obtained from NIBSC, were used for SRD. For lethal IV challenge, Balb/c mice were challenged with the mouse-adapted A/California/7/09 (H1N1)pdm09 strain.

### 2.3. Study Design, Immunization, and Challenge of Mice

The female Balb/c mice (16–18 g) were purchased at the Stolbovaya Mouse Farm of the State Biomedical Technology Center of the Russian Academy of Medical Sciences. The mice were housed in the vivarium of the Influenza Institute of the Health Ministry of the Russian Federation under ad libitum feeding per relevant in‑house animal care guidelines. Five groups of mice were immunized with SGF, U3, U4, VG, and SGU vaccines. The control group intramuscularly received NaCl 0.5 mL 0.9% NaCl (Renewal Pharm Co., Novosibirsk, Russia). Mice were immunized intramuscularly with one dose of a vaccine or phosphate-buffered saline (PBS), after two weeks there was the second immunization with one dose of a vaccine or PBS. The detailed information about the vaccination protocol is represented in Tables S1 and S2. The study was approved by the bioethics committee of the Smorodintsev Influenza Institute №19 from 11 March 2020.

Immunogenicity follow-up lasted for 28 days (Table S1). The following parameters were recorded: antibody titers in HAI and ELISA on day 15 and 28; MN on day 28, and NA-ELISA on day 28. The mice were infected on day 29. The protection potency follow-up lasted for 14 days for the lethal IV model (Table S2). The percentage of surviving animals was recorded on study day 43, while animal body mass was recorded on days 29 to 43. Challenge virus titers were tested in lungs on day 3 after infection, i.e., day 32 after immunization.

For lethal infection, the Balb/c mice were challenged intranasally with 10LD50 of the mouse-adapted A/California/7/09 (H1N1) pdm09 strain. The protective effect of IIV was measured daily by body weight loss and survival rates over a post-challenge period. The control mice were used as a negative control in the challenge studies. For sublethal infection, we used IV strains with 10MID of A/Brisbane IVR-190 (H1N1) and A/Kansas NYMC X-327 (H3N2); with 100MID of Influenza B/Maryland NYMC BX-69A (Victoria lineage), and Influenza B/Phuket/3073/2013 (Yamagata lineage). All the viruses were administered intranasally as 50 μL/mouse following inhalation anesthesia (2–3% isoflurane, 30% O_2_, 70% N_2_O).

The mice were routinely euthanized by placing them in a CO_2_ euthanasia chamber (Vet Tech Solutions). Mouse sera were received as described above [17].

### 2.4. Replication of Influenza Viruses in Lungs

Lungs were harvested from mice as described above [18]. The titers of viruses reproducing in mice lungs were determined as set forth in [17]. Briefly, the virus titers were determined by reproduction on the MDCK cell line using the hemagglutination assay with 1% of chicken RBC. The titer was taken as a value opposite the decimal logarithm of the highest virus dilution rate showing a positive HA response. Virus titers were expressed as a lg 50% tissue infected culture dose (TCID50). 

### 2.5. SRD Assay

The single-radial-immunodiffusion (SRD) assay was performed according to the European Pharmacopoeia and as described above [19]. Briefly, the slides were covered with agarose (Amresco, USA) IV-specific antiserum, diluted as the standard antigen, with the vaccine samples having been mixed, incubated, and prepared following the relevant decline of the dilution rate and the product standards, i.e., 0.75 (sample: buffer = 3:1), 0.5 (1:1) and 0.25 (1:3), respectively. Twenty µL of each diluted portion was poured into the gel cells, including the whole portion. At least two replicates were made for each sample and standard, with some standard dilutions present on each slide. The slides were incubated and stained with 0.3% Coomassie G-250 (Dia-m, Russia). The precipitation area diameter was determined after scanning the slides in the Paint.net graph editor in two perpendicular directions, and the average diameter was calculated. 

### 2.6. HAI Assay

HAI was performed following the relevant WHO protocol [4]. Briefly, mouse sera from the immunized and control groups were treated with receptor destroying enzyme (RDE II, Denka Seiken, Japan) (1:3 titration and kept at 37 °C for 18 h, then heated in a water bath at 56 °C for 30 min). RDE-treated serum was two-fold serially diluted: from 1:10 to 1:1280 on PBS (Biolot, Russia). Fifty µL of the standardized IV antigen (4 HAU) was added to each diluted portion. The slide panels were shaken and incubated for 1 h at room temperature. Control was set up for each serum to exclude the presence of hemagglutinins in chicken RBCs, and to control spontaneous RBC agglutination (PBS). A 1.0% chicken RBC suspension was used to track the reaction. Reaction results were recorded after RBC sedimentation in the control plate cells after 30 to 40 min. The HAI titer of the antiserum is represented as the reciprocal of the highest dilution in which RBC agglutination occurs. In calculating the geometric medium titers (GMT), individual < 1:10 dilutions were taken as 1:5.

### 2.7. MN Assay

Neutralizing antibodies to IIVs were detected in the individual sera of immunized and control animals by MDCK cell culture microneutralization. The MDCK culture, grown in 96‑well culture plates in a CO^2^ incubator at 37 °C to reach a 90–95%-thick monolayer, was washed twice with PBS. Sera were diluted with RDE in a 1:3 ratio and kept at 37 °C for 18 h and then heated in a water bath at 56 °C for 30 min. The sera were controlled individually. Two-fold serum dilutions were performed sequentially (starting from 1:20) in a 100 µL of Dulbecco’s Modified Eagle Medium (DMEM, Biolot, Russia). Each diluted serum portion received 100 µL of diluted virus (PPB) 100 TCID50 on a DMEM medium containing TPCK-treated trypsin (Sigma, USA) as 4 µg/mL and BSA (V fraction) (Biolot, Russia) to reach 0.2% of the end concentration. The mixes were gently stirred and incubated for 1 h at room temperature. Control was set up for each serum to exclude the ability of RBC agglutination (serum + RBC), and to control spontaneous RBC agglutination (PBS + RBC). Then, 100 µL of the mixture was added to each plate cell as a monolayer (double-repeat investigation). The plates were incubated in a CO2 chamber at 37 °C for 72 h. The effect of neutralizing IV reproduction with mouse sera was evaluated by HA reaction as described by F. Sicca et alia [20], as amended. Briefly, at the end of incubation, 50 μL of culture fluid was removed from each plate cell and transferred to U-shaped titration panels. Each cell received 50 µL of 1% chicken RBC suspension. The reciprocal of the last serum dilution preventing agglutination was taken as the neutralizing antibody titer. In calculating GMT, individual <1:20 dilutions were taken as 1:10.

### 2.8. ELISA

High-adsorption 96-well plates (Greiner, Austria) were used to absorb inactivated IV/1 µg/mL or purified A and B influenza virus NA/3 µg/mL (see 2.9) from the study vaccine composition and kept overnight at 40 °C. The plates were treated with a blocking buffer (0,01M PBS, pH 7.2–7.4 (Amresco, USA) with 5% Fetal Bovine Serum (FBS, Biolot, Russia) for 1 h at room temperature and washed three times PBS with TWEEN-20 (Sigma, USA). The plate cells received 100 µL each of the double-diluted sera of the immunized and control mice in the blocking buffer, incubated for 1 h at room temperature. Polyclonal goat anti-mouse IgG, including subclass IgG1, IgG2a (Abcam, UK), marked with horseradish peroxidase, were used as conjugates; 3,3′,5,5′-tetramethylbenzidine (TMB, Biolegend, USA) was used as substrate; the reaction was recorded at 450 nm wavelength. The reciprocal of the highest serum dilution that had an optical density not less than 2 times the average value of the blank was taken as the titer.

### 2.9. Neuraminidase Isolation

The A N1, N2 IV NA, and B IV NA were isolated from the working seed lot of IV monovaccines from the study set. The NA was purified by using the gel “negative” affinity chromatography. The virus-containing fluids of each vaccine strain received 20% Triton X-100 to reach a 2% end point, 500 mM EDTA solution to reach a 2 mM endpoint, and 1 mM DTT solution to reach a 10 mM end point. The resulting mixtures were incubated for 2 h at +37 °C. Chromatographic purification was performed on an AKTA pure 25 GE Healthcare. A 120 mL chromatographic column HAIPrep 16/60 Sephacryl S-500 HR GE Healthcare was washed sequentially with 120 mL of deionized water and 240 mL of 0.1% TWEEN-20 PBS at 1 mL/min. flow rate. Next, 5 mL of the material was put in a column using a Superloop 50 dynamic loop and eluted with a 0.1% TWEEN-20 PBS buffer at 0.5 mL/min flow rate. The process was monitored with a UV flow detector (2mm optical cell route, 280 nm wavelength). During elution, 2.5 mL fractions were collected using an F-9R automatic collector. Fractions obtained during chromatography were analyzed to control the protein mixtures obtained in sodium dodecyl sulfate-polyacrylamide gel electrophoresis (SDS-PAGE) reducing conditions [21,22]. SDS-PAGE was carried out as described earlier [17]. Briefly, 1 µg samples were loaded on the gel pathway (TGX Stain-Free™ FastCast™ Acrylamide Kit, 12%, BioRad, USA), including 2 µg of the molecular mass marker (Precision Plus Protein™ Dual Xtra Prestained Protein Standards, BioRad, USA). The sample was stained with the Coumassie G-250 colloid solution. To remove HA residues, a repeat chromatography was performed to purify the material in 1 mL HAITrap NHS-Sepharose HP GE Healthcare columns containing immobilized monoclonal antibodies to HA of the respective A and B IV strains. 

Next, a mass-spectrometer analysis was used to identify IV-specific proteins, as described earlier [23]. Briefly, polyacrylamide gel (PAG) fragments, presumably containing NA, were cut out, washed free from the stain, and dehydrated in acetonitrile. Then, 2 µL of trypsin (Promega, 20 µg/mL in 50 mM NH_4_HCO_3_) was added to the gel fragments, followed by enzymic hydrolysis at 37 ℃ for 5 h. The medications obtained were mixed with α-cyano-4-hydroxycinnamic acid (HCCA, Bruker) matrix, put on the GroundSteel target and studied on the Matrix-Assisted Laser Desorption Ionization Time-of-Flight Mass Spectrometry (MALDI-TOF/TOF) UltrafleXtreme (Bruker) mass spectrometer in a reflex mode for detecting positive ions. IV-specific proteins were identified using MASCOT (www.matrixscience.com), while referring to the NCBI database (www.ncbi.nlm.nih.gov). Protein identification was considered reliable if the score exceeded the *p* < 0.05 threshold. 

### 2.10. Dot Blotting

For Dot blotting, a Biorad nitrocellulose membrane was cut into 2 × 2 cm squares. Marks were made with a slate pencil to put on 2 µL samples according to the assay protocol. The membrane was left until it had dried up completely. Then, it was blocked in a 5% BSA in PBS + 0.05% TWEEN20 for one hour while being swung. The membranes were incubated for one hour while being swung in a primary antibody solution (Influenza A virus H3N2 HA (Hemagglutinin) antibody [AT1B7], Influenza A virus H1N1 HA antibody [C102], Influenza B Virus HA antibody [10B8], Influenza A virus H1N1 NA (Neuraminidase) antibody [GT288], Influenza B Virus NA antibody [603], GeneTex, USA) in PBS + 0.05% TWEEN20 + 1,5% BSA (Amresco, USA). Then, the membranes were flushed 3 times for 5 min each in PBS + 0.05% TWEEN20. They were incubated later in a secondary antibody solution (Goat anti-Mouse IgG (H + L) Cross-Adsorbed Secondary Antibody, Invitrogen, USA) in 1/1000 PBS + 0.05% TWEEN20 + 1,5% BSA. Then, the membranes were flushed 3 times for 10 min and once for 5 min in PBS + 0.05% TWEEN20. The membrane was stained in TMB (1-Step™ Ultra TMB-Blotting Solution, ThermoFisher, USA) for 15 min.

### 2.11. Statistics

The statistical significance of differences in antibody titers was estimated using GraphPad Prism v6.0. Statistical difference in body mass dynamics was calculated using ANOVA to identify differences between the IV-specific mouse groups, or on specific days using GraphPad Prism v6.0. Survival rates were compared between various groups by building Kaplan–Meier curves in GraphPad Prism v6.0. Differences were considered significant at *p* < 0.05.

## 3. Results

### 3.1. Determining Vaccine-Specific Activity in SRD

The normative HA content (based on the information from vaccine inserts) in U3, U4, and VG vaccines, and the results of determining HA content in SRD, are presented in Table 1. The HA content in A(H3N2) IV and B/Victoria IV in U3 was about the normative content, while that in A(H1N1) was slightly higher than the label amount. In the U4 vaccine, the HA content of all vaccine strains matched with the standard value, but was significantly higher (15 µg/dose) for all the three viruses in VG; most significant were the A(H1N1) differences, where HA was more than double the norm. 

It is impossible to determine HA content in SGF and SGU vaccines due to the presence of a synthetic polymer in the vaccines. Here, the normative HA content in SGF and SGU vaccines is 5 µg for A(H1N1), 5 µg for A(H3N2), and 11 µg/mL for B/Victoria.

### 3.2. Determining Immune Response in HAI Assay

**Figure 1:**
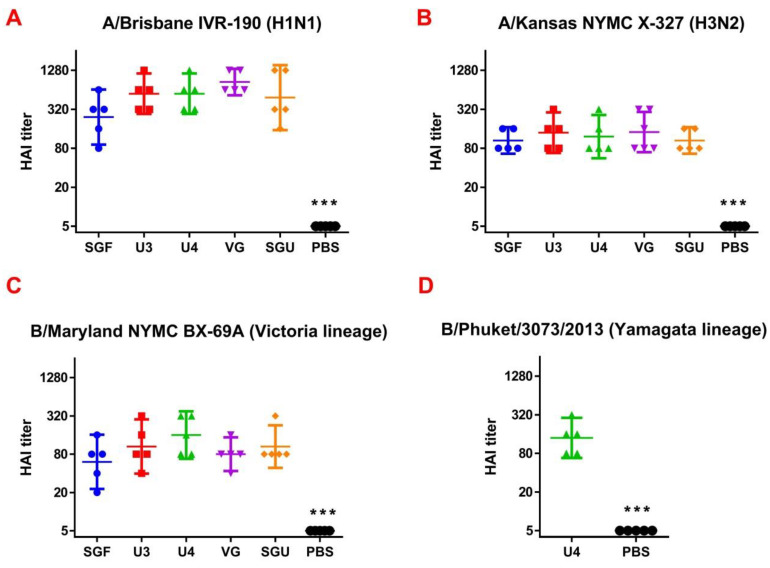
The hemagglutination inhibition (HAI) titers after the second immunization represented for (**A**) A/Brisbane IVR-190, H1N1 (*n* = 5/group); (**B**) A/Kansas NYMC X-327, H3N2 (*n* = 5/group); (**C**) B/Maryland NYMC BX-69A Victoria lineage (*n* = 5/group); (**D**) B/Phuket/3073/2013 Yamagata lineage (for the U4 quadrivalent vaccine only) (*n* = 5/group). Data are presented as individual titers and GMT (horizontal line). *** difference from immunized groups (*p* < 0.0001).

The IV antibody titers in HAI on day 29 are presented in Figure 1. The titers were significantly higher in all study groups versus control after the second immunization. After the first immunization (data not shown), they were slightly higher or equal to those of the control. No significant difference was found in the anti‑HA antibody titers in HAI between the mice groups immunized by various IIVs. 

### 3.3. Determining Immune Response in MN Assay

**Figure 2:**
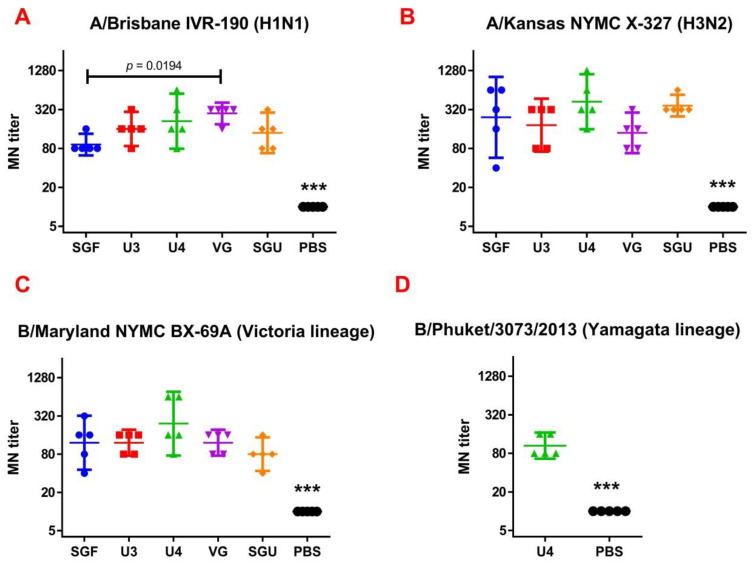
The results of the micro-neutralization (MN) assay represented for (**A**) A/Brisbane IVR-190, H1N1 (*n* = 5/group); (**B**) A/Kansas NYMC X-327, H3N2 (*n* = 5/group); (**C**) B/Maryland NYMC BX-69A Victoria lineage (*n* = 5/group); (**D**) B/Phuket/3073/2013 Yamagata lineage (for the U4 quadrivalent vaccine only) (*n* = 5/group). Data are presented as individual titers and GMT (horizontal line). *** difference from immunized groups (*p* < 0.0001).

The MN antibody titers to IV strains on day 29 are presented in Figure 2. The MN antibody titers were significantly higher in all study groups versus control after the second immunization. No significant difference was found in the neutralizing antibody titers in MN between the mouse groups immunized by various IIVs.

### 3.4. Neuraminidase Isolation

**Figure 3:**
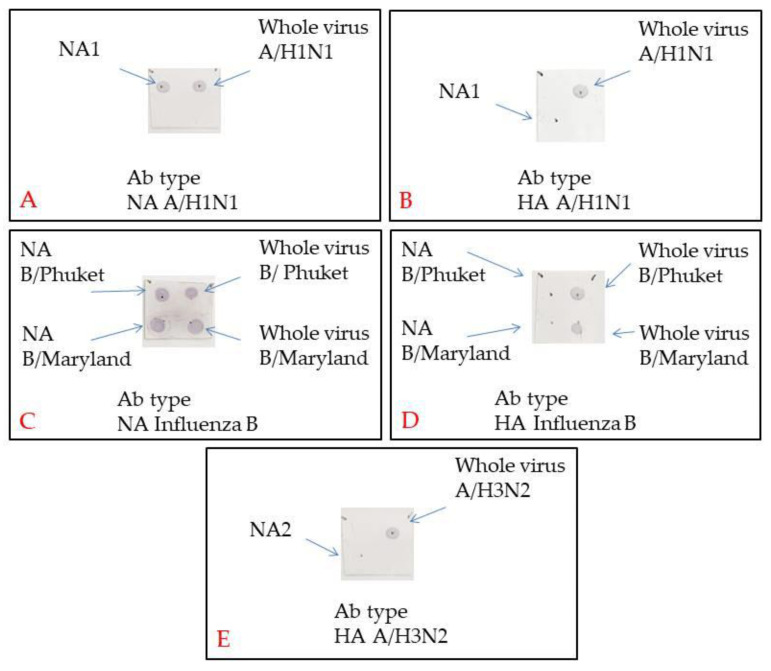
Dot blotting assay for the pure NA product. Represented are the membranes treated with (**A**) Influenza A virus H1N1 NA antibodies; (**B**) Influenza A virus H1N1 HA antibodies; (**C**) Influenza B Virus NA antibodies; (**D**) Influenza B Virus HA antibodies; (**E**) Influenza A virus H3N2 HA antibodies.

The protein preparations enriched in NA and depleted in other major viral proteins were obtained by the sequential chromatography sessions with samples of the monovalent vaccine working seed lots. Figure S1 shows the NA product chromatograms. NA was present in Fraction 3 of all the products. The fractions gathered were subjected to SDS‑PAGE under reducing conditions. NA-containing fractions are shown in Figure S2. To identify IIV-specific proteins by MALDI‑TOF/TOF, 3 to 4 bands were cut out from the gel, which presumably contained NA. The latter was confirmed in the PAG bands of 65 to 70 kDa (Figure S2). Apart from NA, the first chromatography session also detected nucleoprotein from influenza virions and HA in the samples. The results of mass spectrometry of NA in monovaccines are presented in Table S3. So, IV NA corresponding to the virus type was determined in all preparations. To deplete the HA‑containing protein products obtained, a second chromatography session was performed using immobilized monoclonal antibodies. The final NA‑containing products obtained were subjected to SDS-PAGE to check for the absence of proteins in the range of 20–30 kDa and over 75 kDa (data not shown). The Dot Blot results (Figure 3) show the presence of NA and the absence of HA in the respective preparations and their suitability for follow-on NA-ELISA.

### 3.5. Determining NA Immune Response in ELISA

**Figure 4:**
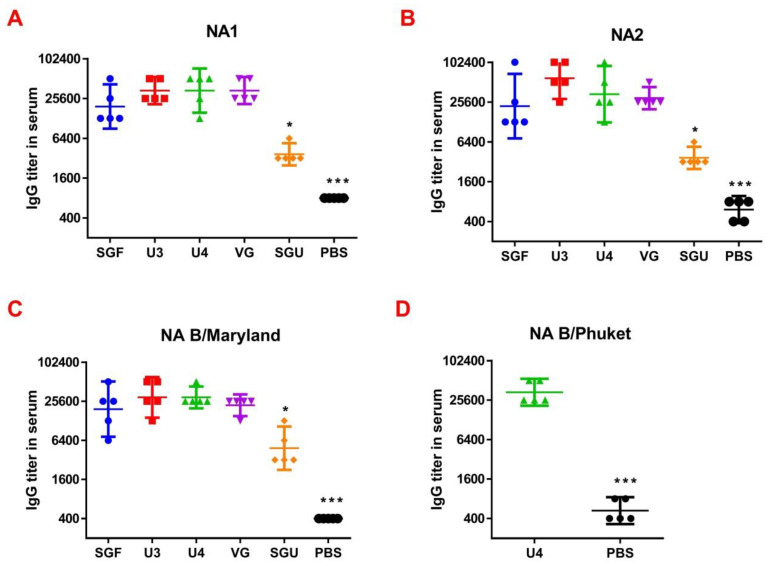
The results of the neuraminidase enzyme-linked immunosorbent assay (NA-ELISA) assay represented as (**A**) the anti-NA1 IgG titers for A/Brisbane IVR-190, H1N1 (*n* = 5/group); (**B**) anti-NA2 IgG titers for A/Kansas NYMC X-327, H3N2 (*n* = 5/group); (**C**) anti-NA IgG titers for B/Maryland NYMC BX-69A Victoria lineage (*n* = 5/group); (**D**) anti-NA IgG titers for B/Phuket/3073/2013 Yamagata lineage (for U4 the quadrivalent vaccine only) (*n* = 5/group). Data are presented as individual titers and GMT (horizontal line). * difference from other immunized groups with *p* ≤ 0.01, *** difference from all immunized groups with *p* ≤ 0.0001.

Following the second immunization, ELISA and the enriched with NA protein preparations were used to determine the NA-specific IgG content in mice in the experimental and control groups (Figure 4).

It was found that after the second immunization, anti-NA IgG titers significantly differed from the control in all the vaccinated mouse groups. The lowest levels of anti-NA IgG (GMT = 32,000–48,502) to the purified A/H1N1, A/H3N2, B/Victoria lineage, and B/Yamagata lineage were detected in SGU‑immunized mice—significantly lower than in all other study groups. No significant difference was reported in the antibody titers to NA in the mice immunized with SGF, U3, U4, and VG vaccines.

### 3.6. Determining IgG and Specific IgG1 and IgG2a Subclasses in ELISA after Second Immunization

**Figure 5:**
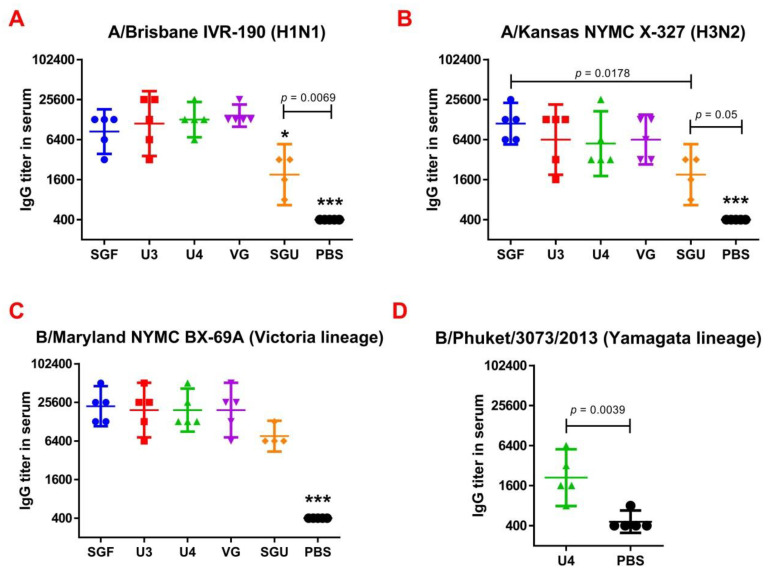
The results of the ELISA assay after the second immunization represented as (**A**) IgG titers for A/Brisbane IVR-190, H1N1 (*n* = 5/group); (**B**) IgG titers for A/Kansas NYMC X-327, H3N2 (*n* = 5/group); (**C**) IgG titers for B/Maryland NYMC BX-69A Victoria lineage (*n* = 5/group); (**D**) IgG titers for B/Phuket/3073/2013 Yamagata lineage (for U4 the quadrivalent vaccine only) (*n* = 5/group). Data are presented as individual titers and GMT (horizontal line). ). * difference from other immunized groups with *p* ≤ 0.01, *** difference from all immunized groups with *p* ≤ 0.0001.

After the second immunization, ELISA was used to determine the whole-virus specific IgGs as well as IgG1 and IgG2a subclasses in the experimental and control mouse groups (Figure 5).

Following the second immunization, for all studied vaccines, anti-HA IgG titers to type A and B viruses differed significantly from the control. However, the lowest titers were found in SGU-immunized mice, which was significantly lower than in all other study groups. No significant difference was observed in IgG titers in the mice immunized by SGF, U3, U4, and VG. In U-4-immunised mice, the IgG level to B/Phuket/3073/2013 (Yamagata lineage) was also estimated. After the second immunization, GMT IgG was 2 111.2—significantly different from the control group (*p* < 0.05).

**Figure 6:**
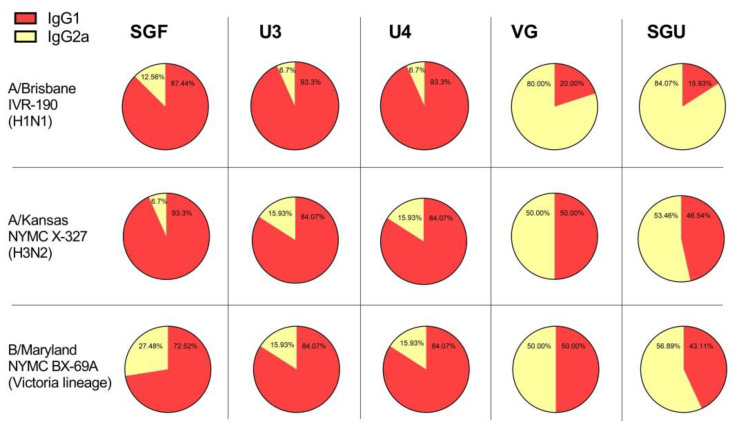
The IgG1/IgG2a ratio after the second immunization.

On day 14 following the second immunization, both IV-specific IgG subclasses were detected in mice, which indicate a mixed Th1/Th2 immune response profile, Figure 6. However, the IgG1/IgG2a ratio varied significantly between vaccines. For instance, SGF, U3, and U4 induced the highest IgG1/IgG2a ratio with a trend toward a Th2 profile, IgG1/IgG2a ratio varied from 2.64 to 27.86. At that, VG and SGU induced the lowest IgG1/IgG2a ratio in favor of a mixed Th1/Th2-type response with a trend toward a Th1 profile, IgG1/IgG2a ratio varied from 0.2 to 1.0. In U4-immunised mice, it was not possible to reliably determine the IgG1/IgG2a ratio in relation to B/Phuket (data not shown).

### 3.7. Estimating the IIV Protective Effect on a Lethal IV Infection Model

**Figure 7:**
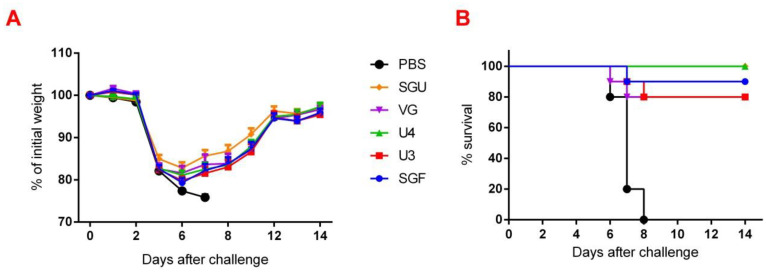
The protective effect of IIV assessed on a lethal infection model for A/California/7/09 (H1N1)pdm09. Represented are (**A**) Weight loss values for experimental and control (injected with PBS buffer) mouse groups (*n* = 10/group; *p* < 0.0001); (**B**) The survival rates in both groups (*n* = 10/group; *p* ≤ 0.0012).

Changes in body mass and mortality of immunized and control mice after the former were infected with A/California/7/09 (H1N1)pdm09 virus strain are shown in Figure 7. The maximum weight loss was reported by the control group—22.7%—which was significantly higher than in the immunized mice. The maximum weight loss in the immunized was recorded on day 6 after infection, while decreasing till day 8 in the control. The SGU and U4 vaccines showed 100% protection of the mice against the lethal A/California infection (Figure 7). The SGU, U3, and VG vaccines secured an 80–90% protection rate.

### 3.8. Estimating the IIV Protective Effect in IV Reproduction in the Lungs

**Figure 8:**
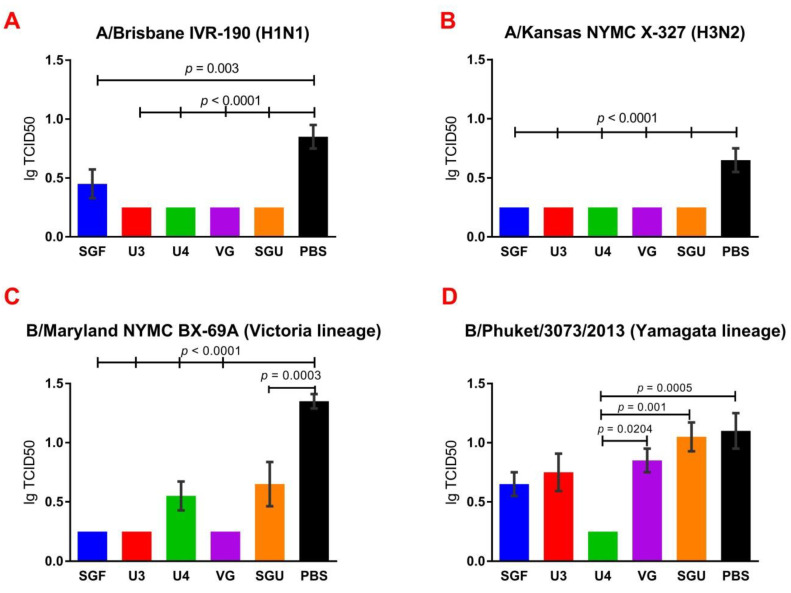
IV reproduction in the lungs of the sublethally infected and control mice (*n* = 5/group). Represented are virus titers in the lungs after infection with (**A**) A/Brisbane IVR-190 (H1N1); (**B**) A/Kansas NYMC X-327 (H3N2); (**C**) B/Maryland NYMC BX-69A Victoria lineage; (**D**) B/Phuket/3073/2013 Yamagata lineage.

Overall, all the IIVs confirmed their protective effect in IV reproduction in mice lungs against homologous IVs on day 3 after sublethal infection with the IIVs listed. The lung-related IV reproduction results are presented in Figure 8. The data obtained show that reproduction of A/Brisbane IVR-190 (H1N1), A/Kansas NYMC X-327 (H3N2), and B/Maryland NYMC BX-69A in the immunized mice lungs were significantly lower than in the control group (*p* ≤ 0.003). Reduced viral replication of B/Phuket/3073/2013 was found only in the U4-immunised mice, with a significant difference from that of the control (*p* = 0.0005) due to the strain composition of this quadrivalent vaccine. There were no significant differences in the replication of A/Brisbane IVR-190 (H1N1), A/Kansas NYMC X-327 (H3N2), and B/Maryland NYMC BX-69A between the mice groups immunized with various IIVs.

## 4. Discussion

Inactivated influenza vaccines are in demand in Russia and around the world. They account for 80% of the global market. However, the protective properties, efficacy, and nature of the immune response of commercial vaccines registered in Russia have not been investigated adequately so far. In this work, we have used a set of some routine methods and proposed new approaches for analyzing the immunogenicity of the vaccines included in the Russian National Immunization Program.

Following the results of the determination of the HA content in IIVs obtained by SRD and normative documentation, the SGF/SGU vaccine group differs from the U3/U4 vaccines by 3 to 4 times, and from the VG vaccine by 6 times. Despite this fact, the standard methods, such as HAI and MN, did not show any statistically significant difference in the immune response measured in mice immunized with various IIVs. All tested samples of animal sera after immunization had a significant deviation from the non-immunized control and showed sufficient levels of the production of neutralizing antibodies and the HA inhibition efficiency measured in HAI assays. Surprisingly, they did not differ from each other (Figure 1 and Figure 2). Similar conclusions were made from the experiments in a lethal infection model. After the mice had been infected with the lethal A/California/7/09 (H1N1) pdm09AM virus strain, all the studied vaccines showed a protective effect, which was confirmed by a decrease in mouse weight loss and an increase in survival rate, with a significant difference from the control mouse groups injected with a buffer instead of vaccine preparation (Figure 7). Although some vaccines showed a lower protection level in the lethal infection experiments, this may be due to little statistical sampling or an incomplete match in antigenic determinants detected between the 2018 and 2009 viruses, as described earlier [24]. An experiment to study virus reproduction in sublethal infection showed that all the immunized mice had a low homologous virus reproduction titer and a statistically significant difference from that of the control (Figure 8). Thus, although the evaluation techniques applied managed well in distinguishing immunization effects from that of a placebo, they are not very informative to reveal peculiarities of immune responses provided by various commercial vaccines prepared in a similar way (within the same vaccine type, IIVs) and did not show a dependency on the HA content in a dose.

In an attempt to more deeply understand the difference in the protective immune responses elicited by the vaccines in mice, we used ELISA and NA-ELISA approaches in addition to the standard methods. It has been repeatedly shown that the recipient’s immune response to NA is much lower than that to HA [25]. However, several cases of the essential contribution of NA into the immunological defense against infection have also been published [26,27]. Thus, in BALB/c mice immunized with a single dose of neuraminidase-expressing DNAs by electroporation stable long-term immunity against homologous IV was developed [28]. It was also found that in comparison with the anti-HA antibodies the anti-NA antibody pool boasts essential heterosubtypic protection in the BALB/c mice against a lethal challenge with 2009 H1N1pdm or H5N1 virus [29]. Further, a study conducted by Couch et al. to compare the immune responses to HA and NA using the commercially available trivalent vaccines reported that IIVs with a similar HA content per dose induce a similar anti-HA immune response in healthy adults, while some vaccines elicited a better immune response to N1 and N2 subtype NA proteins than the others. At the same time, an intranasally administered modified live attenuated vaccine (LAIV) has been found to elicit a poor systemic antibody response to both HA and NA compared to IIVs. [30]. Based on the above evidence, the issue of assessing immune response to NA when analyzing the quality of vaccines is becoming a pressing matter.

Apart from ELISA, an enzyme-linked lectin assay (ELLA) and neuraminidase inhibition enzyme-linked lectin assay (NI-ELLA) are often used to measure immune responses to NA. ELLA and NI-ELLA are enzyme immunoassays based on the viral NA enzymatic activity with regard to sialic acid and the blocking properties of the antiserum containing antibodies to NA [31]. However, NA often loses its enzymatic activity during production of IIVs. According to Monto, only about 37% of recipients developed NI immune response when immunized with trivalent IIVs, versus 77% and 67% for HAI and MN, respectively [25].

So, we have suggested the method of enriching monovalent bulks from the list of seasonal strains with the NA protein followed by NA-ELISA. This approach is based on the direct interaction of NA protein and anti-NA antibodies derived from immunized animal serum. It does not depend on the enzymatic activity of NA itself. Moreover, each NA type/subtype-enriched preparation was used for the reaction with its own bulk strain. This eliminated the problem of mismatch of virus strains. As a result, when measuring the ELISA and NA-ELISA immune responses, the data obtained showed a significant difference between the investigated vaccines. The NA-ELISA data (Figure 4) showed not only a significant difference between the studied vaccines and the controls but also revealed significant differences between the studied vaccines. For instance, the immune response to NA was the lowest when the SGU preparation was used for immunization. Similarly, the lowest IgG titers were detected in ELISA after the second immunization of mice with SGU among all vaccines studied (Figure 5).

Surprisingly, the SGF vaccine, which has an identical formulation with the SGU vaccine in terms of composition and antigen content, has shown some NA-ELISA titer values similar to those elicited by the U3, U4, and VG vaccines. Yet, SGU demonstrated essentially wider data distribution compared to U3, U4, and VG. That probably means instability of its response. The ELISA results for SGU and SGF are most likely down to the lowest content of the antigen and other viral proteins within a vaccine dose. Thus, it was shown earlier that the immune response to NA is the direct function of the antigen quantity, which is most clearly manifested in high-dose immunization [30,32].

Another interesting point of our investigation concerns the detection with ELISA of different values of IgG1 and IgG2a subclass titers after second immunization of mice with various vaccines. As has been repeatedly shown previously, experimental infection with influenza virus and immunization with LAIVs leads to the formation of a mixed Th1/Th2 immune response in BALB/c mice with a predominance of the IgG2a subclass antibodies [33,34,35]. Likewise, whole-virion IIVs also develop a mixed Th1/Th2 response with an increased IgG2a antibody level [34,35,36]. At the same time, after the immunization of BALB/c mice with split or subunit IIVs, either the IgG1 antibodies prevail that directly neutralize the virus, or antibodies of IgG2a subclass, whose action mechanism is associated with antibody-dependent cellular cytotoxicity [37,38].

Both the IgG1 and IgG2a antibodies were shown to ensure protection against lethal IV challenge [39]. However, the immune response profile and, accordingly, the ratio of different IgG subclasses after the immunization with IIVs, might be influenced by a number of factors, including protein composition of the vaccine, adjuvants and some other extra substances included in the vaccine product [40,41,42]. Besides, the presence of conservative viral proteins, such as M1, M2, and NP in the vaccine has been found to affect the IgG1/IgG2a ratio [43]. In our experiments, the IgG1 subclass predominated in animals immunized with U3, U4, and SGF vaccines, while the calculated IgG1/IgG2a ratio was close to 1, or sometimes IgG2a prevailed, in the VG- and SGU-immunized animals. We surmise that higher activity of T-helper 2 (Th2) and, accordingly, the production of neutralizing antibodies is more pronounced when the animals were immunized with U3, U4, and SGF vaccines, while a higher activity of T-helper 1 (Th1) and antibody-dependent cellular cytotoxicity apparently plays an important role in the case of response to VG and SGU. Importantly, despite similar IgG1/IgG2a ratios calculated for SGU and VG immunization, the quantitative response to these vaccines was significantly different: it was pronounced for VG but was several orders of magnitude lower for SGU.

It is most likely that the essential difference in the IgG1/IgG2a ratios measured upon BALB/c vaccination with various IIVs is associated with the differences in their protein compositions, which we demonstrated earlier using label-free quantitative mass spectrometry [44]. That study manifested that the IIVs showed interpretable results only with respect to the abundance of the major antigen proteins, HA and NA, while there was a fairly wide range of data for the core proteins present in minor proportion within the investigated IIVs. That said, the IgG1/IgG2a ratios for BALB/c IIV immunization might be linked to protein composition, degree of purification of the virus-containing fluid during vaccine production, and contingent on the number of core proteins remaining in the final formula. The role of certain unsplit virions present in some amount in a number of vaccine products cannot be ruled out. However, a more in-depth statistical analysis is required for reliable assessment of this factor.

## 5. Conclusions

The employment standard methods of measuring immune responses to the inactivated influenza vaccines used in the Russian National Immunization Program revealed that all vaccines induce a detectable response in an animal model, which, as it turned out, does not significantly depend on the antigen content in a vaccine dose. However, applying additional immunosorbent assays (ELISA and NA-ELISA; detecting the IgG1 and IgG2a antibodies) disclosed significant differences in the nature of the immune response to various commercial vaccines. It was shown for the first time that U3, U4, and VG, unlike SGU and SGF, induce a more balanced immune response in BALB/c mice, including a good response for both HA and NA. We associate this directly with the antigen content corresponding to WHO guidelines. Further studies on immune response in human sera using an expanded plate would shed light on the nature of the immune response to IIVs of various compositions and help identify which vaccine is better and why.

## Figures and Tables

**Table 1:** Hemagglutinin (HA) content in inactivated influenza vaccines (IIVs) by single-radial-immunodiffusion (SRD) (right column) vs. the norm (left column).

	A(H1N1) Normative/SRD		A(H3N2) Normative/SRD		B/Victoria Normative/SRD		B/Yamagata Normative/SRD	
U3	15.2	17.9	15.1	14.6	13.4	13.5	-	-
U4	15.5	16.0	15.6	16.8	15.7	14.9	15.2	15.9
VG	15	31.3	15	19.0	15	18.2	-	-

## Supplementary Materials

The following are available online at https://www.mdpi.com/2076-393X/8/4/756/s1. Table S1. Immunogenicity study design; Table S2. Protection study design; Table S3. Identification of neuraminidase in influenza virus monovaccines via mass spectrometry; Figure S1. Chromatograms B/Vic, B/Ym, A/N2, and A/N1; Figure S2. SDS-PAGE of B/Vic, B/Ym, A/N2, and A/N1.
