# Supplementary material for: Comparative Immunological Study in Mice of Inactivated Influenza Vaccines Used in the Russian Immunization Program
Source: Vaccines (Basel). 2020 Dec 12;8(4):756. doi: 10.3390/vaccines8040756 (PMC7768547; doi:10.3390/vaccines8040756)

SUPPLEMENTARY MATERIALS

Table S1. Immunogenicity study design

vaccines	mice	Days of the study			
		Immunization		Blood sampling	
		I (day 1)	II (day 15)	day 15	day 29
SGF	15	1 dose	1 dose	5	10
U3	15	1 dose	1 dose	5	10
U4	15	1 dose	1 dose	5	10
VG	15	1 dose	1 dose	5	10
SGU	15	1 dose	1 dose	5	10
PBS	15	0.5 ml	0.5 ml	5	10

Table S2. Protection study design

		Days of the study							
	mice	Immunization		Challenge, day 29					
				Lethal challenge		Sublethal challenge (sampling on day 32 after challenge)			
		I (day 1)	II (day 15)	A/California H1N1pdm09 10LD50	Survey day 29 to day 43	A/Brisbane (H1N1) 10MID	A/Kansas (H3N2) 10MID	B/Maryland 100MID	B/ Phuket 100MID
SGF	30	1 dose	1 dose	10	weight loss and survival rates	5	5	5	5
U3	30	1 dose	1 dose	10		5	5	5	5
U4	30	1 dose	1 dose	10		5	5	5	5
VG	30	1 dose	1 dose	10		5	5	5	5
SGU	30	1 dose	1 dose	10		5	5	5	5
PBS	30	0.5 ml	0.5 ml	10		5	5	5	5

Table S3. Identification of neuraminidase in influenza virus monovaccines via mass spectrometry. In B/Vic (B/Colorado Victoria), B/Ym (B/Phuket Yamagata), and A/N2 (A/Kanzas H3N2) samples, NA was reliably identified, the score exceeded the threshold value. The score for A/N1 (A/Brisbane H1N1) was below the threshold, so it can be assumed that NA is located within this interval. Apparently, this is probably due to the shortage of ions in the spectrum because NA was highly glycosylated.

.

	MW (PAGE)	Similar strains with the respective NA subtype	Score / threshold
B/Vic	~65 kDa	B/Singapore/49H/2010; B/India/P1015910/2010; B/Nairobi/351/2005	78 / 74
B/Ym	~65 kDa	B/Japan/315/2008	117 / 74
A/N2	~70 kDa	A/Beijing/106/2012; A/Louisiana/06/2011; A/Wisconsin/10/2011	78 / 74
A/N1	~70 kDa	A/Wisconsin/09/2013; A/California/10/2014; A/Helsinki/771M/2014	59 / 74

Figure S1. Chromatograms B/Vic, B/Ym, A/N2, and A/N1. Purple color stands for absorption at 280 nm wavelength, while red shows the fractions collected. In all the products, NA was present in fractions No. 3 (see the arrows).

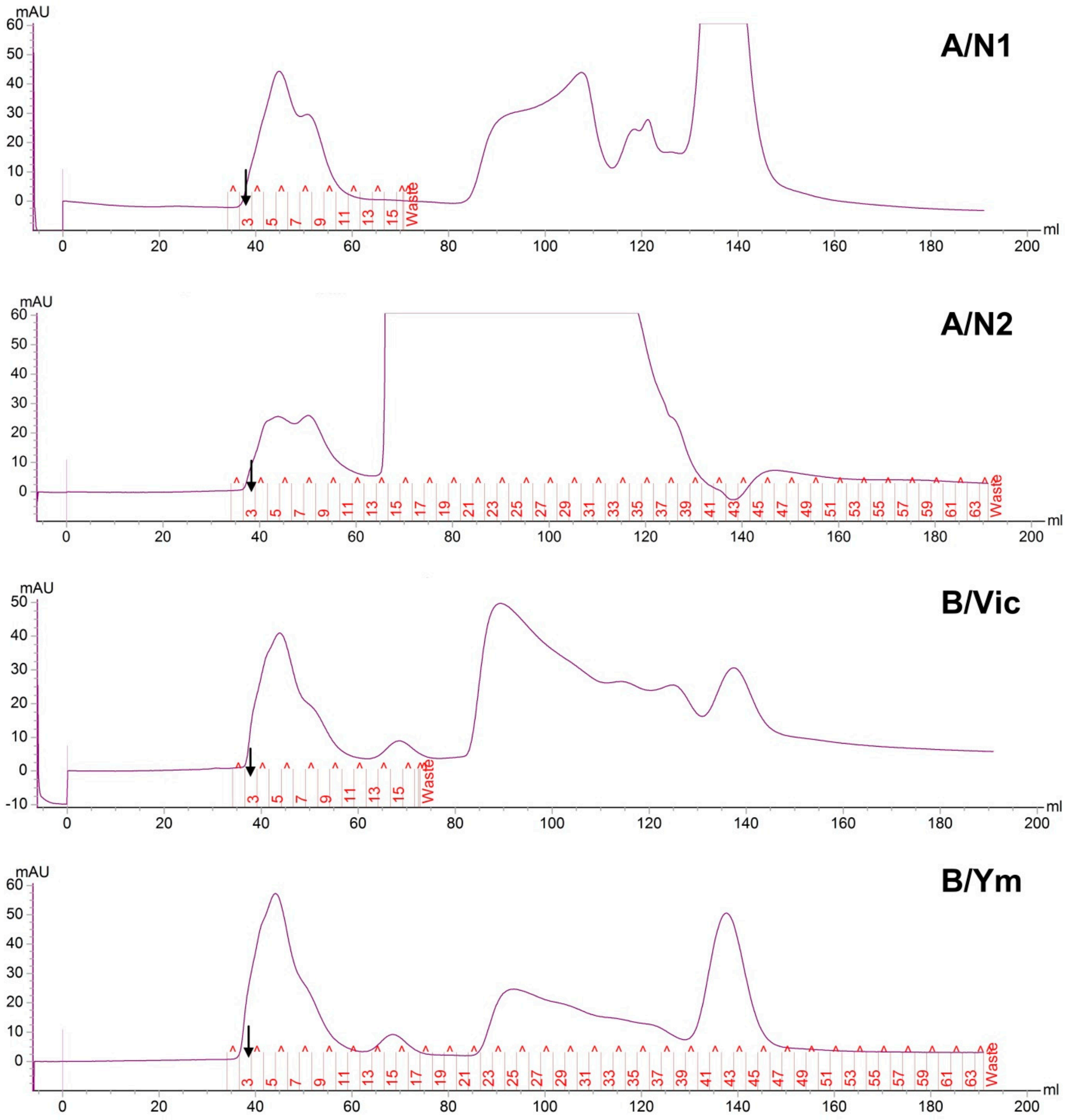

Figure S2. SDS-PAGE of B/Vic, B/Ym, A/N2, and A/N1. M is the molecular-weight size marker. The dots indicate the spots cut out for mass spectrometry analysis. Arrows show the areas where the IV NA was reliably detected. An exclamation mark (!) indicates the spot with the low score / threshold ratio found for A/N1.

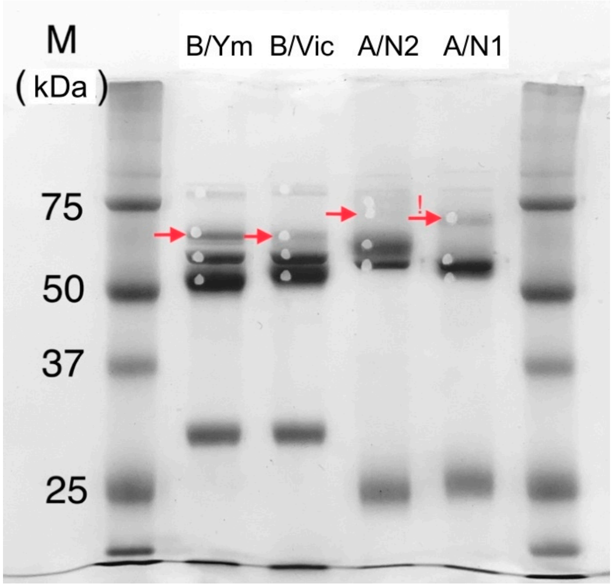

Supplement: Supplementary file 1 [file vaccines-08-00756-s001.pdf]
